# Spinal cord trauma and the molecular point of no return

**DOI:** 10.1186/1750-1326-7-6

**Published:** 2012-02-08

**Authors:** Ping K Yip, Andrea Malaspina

**Affiliations:** 1 Centre for Neuroscience and Trauma, Blizard Institute, Barts and The London School of Medicine and Dentistry, Queen Mary University of London, UK

## Abstract

A mechanical trauma to the spinal cord can be followed by the development of irreversible and progressive neurodegeneration, as opposed to a temporary or partially reversible neurological damage. An increasing body of experimental and clinical evidence from humans and animal models indicates that spinal cord injury may set in motion the development of disabling and at times fatal neuromuscular disorders, whose occurrence is not normally associated with any major environmental event. This outcome appears to be dependent on the co-occurrence of a particular form of mechanical stress and of a genetically-determined vulnerability. This increased vulnerability to spinal cord injury may depend on a change of the nature and of the timing of activation of a number of neuroprotective and neurodestructive molecular signals in the injured cord. Among the main determinants, we could mention an altered homeostasis of lipids and neurofilaments, an earlier inflammatory response and the failure of the damaged tissue to rein in oxidative damage and apoptotic cell death. These changes could force injured tissue beyond a point of no return and precipitate an irreversible neurodegenerative process. A better knowledge of the molecular signals activated in a state of increased vulnerability to trauma can inform future treatment strategies and the prediction of the neurological outcome after spinal cord injury.

## Introduction

Acute or chronic compressive radiculopathies and/or myelopathies are associated with a wide range of transitory or permanent neurological disturbances [1,2]. Less commonly, as a result of these traumatic events, the development and progression of pain, loss of power and muscle wasting can be observed over time. These neurological features are more typical of amyotrophic neuralgias, neuromuscular disorders better known as idiopathic or genetically-induced conditions [3]. Different modalities of neurotraumas have also been linked to the development of either localised muscle wasting (focal amyotrophy), or to the development of a more widespread form of muscle weakness and wasting which become clinically indistinguishable from motor neuron disease (MND), an irreversible and generally fatal neurodegenerative disorder associated with a survival of approximately 3 to 5 years from disease onset and to the loss of motor cells in the cortex, brain stem and spinal cord [4]. Case studies have indicated how amyotrophic lateral sclerosis (ALS), a clinical form of MND, may have a higher occurrence in individuals exposed to hard physical contact, including mechanical traumas to the head, neck or back [5-18]. The potential role of trauma in engendering ALS also emerges in association with other stressors, like bone fractures and surgical intervention [19,20].

From a molecular perspective, the clinical observations reported above suggest that a neurotrauma may mobilise molecular processes leading to a progressive neurodegenerative disorder normally occurring as an idiopathic or genetically-induced condition. The development of a progressive neurodegenerative disorder following spinal cord injury (SCI) may be facilitated by a genetic trait which renders certain individuals more vulnerable to the pre-existence of a sub-clinical degenerative process in the affected tissue, which is more likely to be present with aging and to be made worse and/or precipitated by neurotrauma. The recognition of molecular factors determining the hitherto unforeseen consequences of neurotrauma constitute an important step towards the understanding of neurodegeneration and towards the development of novel treatment strategies and biomarkers. This paper will review the molecular response to spinal trauma and its temporal unravelling, as well as those states known to modulate spinal cord tissue vulnerability to trauma.

### Spinal cord injury: early and late *injury genes *and tissue regeneration

**Figure 1:**
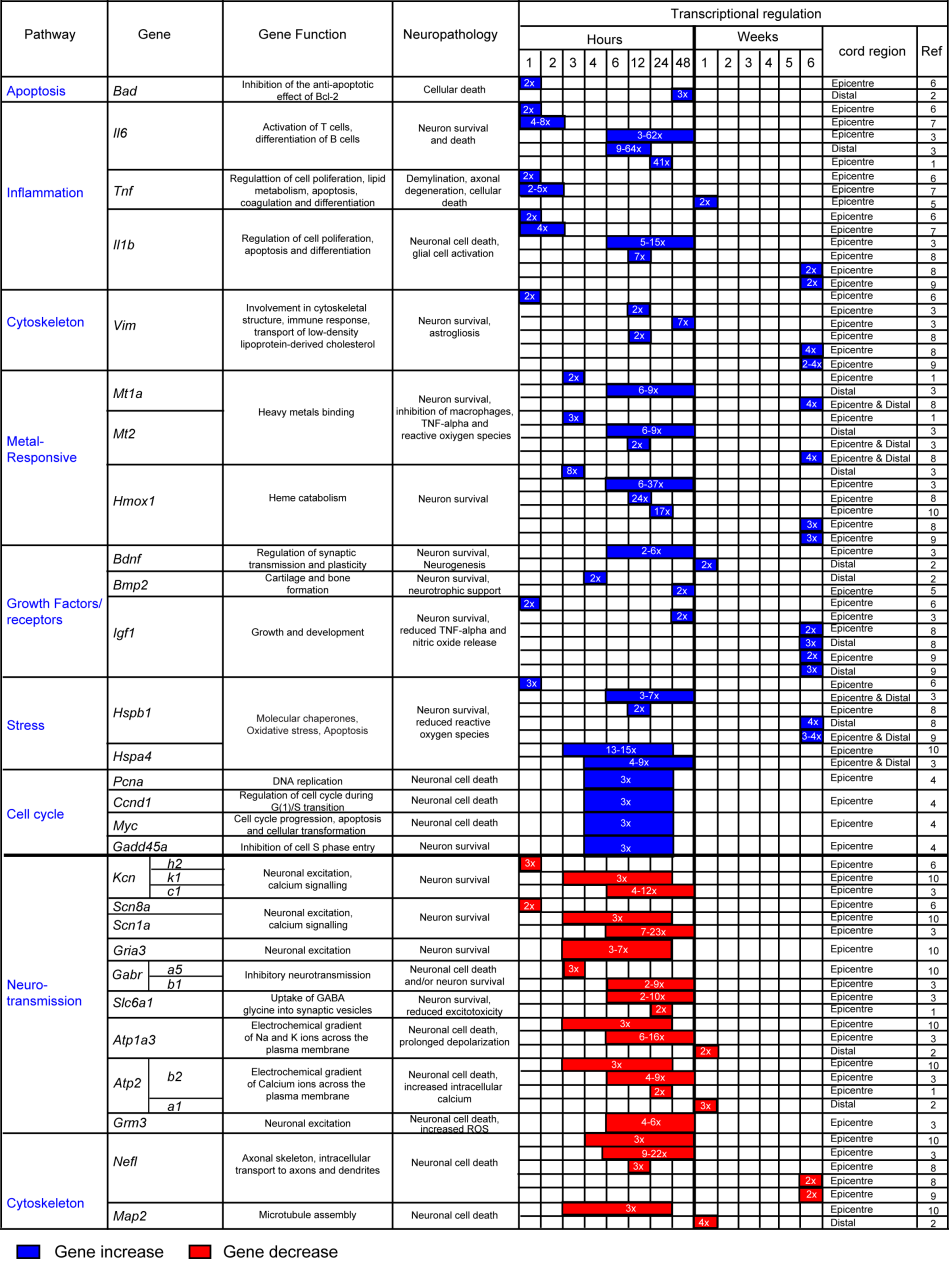
**Differentially regulated genes that become activated or inhibited within the first few hours from injury, reported as early *injury genes***. Examples of molecular responses (pathways) identified in normal rodent spinal cord after mechanical injury, according to the pathway analysis of recent transcriptomic studies of SCI [28,29]. We report information regarding the functional and neuropathological effects that each reported gene may have, based on an overview of published data. The nature of the differential regulation and the location of the transcriptional change with regard to the epicenter of injury are also reported. Blue color indicates an increase in gene expression. Red color indicates a decrease in gene expression. References: 1; Aimone et al., '04, 2; Bareyre et al., '02, 3; Carmel et al., '01, 4; Di Giovanni et al., '03, 5; Malaspina et al., '08, 6; Nesic et al., '02; 7; Pan et al., '02; 8; Resnick et al., '04; 9; Schmitt et al., '06, 10; Song et al., '01.

**Figure 2:**
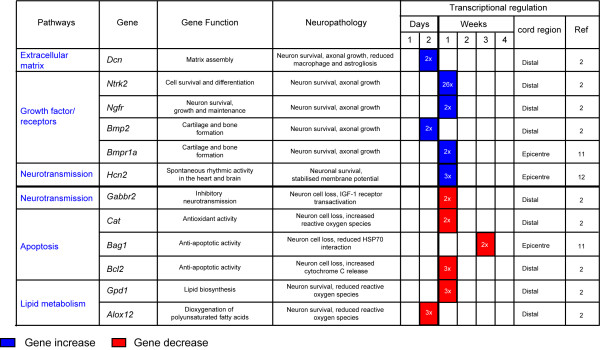
**Differentially regulated genes that appear to have a delayed response which become activated or inhibited more than 2 days from the trauma, reported as late *injury genes***. Examples of molecular responses (pathways) identified in normal rodent spinal cord after mechanical injury, according to the pathway analysis of recent transcriptomic studies of SCI [28,29]. We report information regarding the functional and neuropathological effects that each reported gene may have, based on an overview of published data. The nature of the differential regulation and the location of the transcriptional change with regard to the epicenter of injury are also reported. Blue color indicates an increase in gene expression. Red color indicates a decrease in gene expression. References: 2; Bareyre et al., '02, 11; Fan et al., '01, 12; Jokic et al., '10.

Different molecules and their spatio-temporal activation in the injured tissue may have diverging effects on total cell loss and on tissue regeneration. As such, they determine the outcome of SCI. Mechanical injuries cause necrosis of those neurons directly affected by the force of impact (primary injury phase). A secondary injury phase is characterised by a protracted neuronal loss driven by changes in oxygen, glucose, neuroactive lipids and eicosanoids homeostasis, by the release of free radicals and biogenic amines, endogenous opioids and excitatory amino acids [21-27]. The use of large-scale spinal cord transcriptional analysis in well-established animal models of SCI has shown the rapid differential regulation of a number of genes, here referred to as early *injury genes *(within a few hours from injury), and the slower response of others termed as late *injury genes *(more than 48 hours from injury). Expression profiling of injured spinal cord tissue is a powerful method for unearthing the molecular consequences of trauma, particularly if gene expression changes are considered in light of the associated functional and histopathological alterations. In Figures 1 and 2 we have reported the main molecular responses which have been described in the rat spinal cord following injury, according to recent pathway analyses of gene expression studies and to other relevant transcriptomic studies of SCI [28,29]. Within each molecular pathway, we have selected some of the most representative differentially regulated genes with a very early and a late activation (genes activated in the first few hours from injury and after 48 hours respectively). The figures display the levels of transcriptional regulation and the position of the reported gene expression change with respect to the epicentre of injury, along with the functional role and the neuropathological changes which have been associated with the differential regulation of each gene.

A number of molecular pathways become activated in an early post-SCI phase (less than 48 hours from SCI; Figure 1) [29-39]. This early response, mostly reported at the epicentre of injury, encompasses biological signals and early *injury genes *which have opposite effects on cell survival. Whilst apoptotic and pro-inflammatory responses are likely to be detrimental to cell survival other more protracted growth signals facilitate tissue repair. Among the latter, metallothioneins promote angiogenesis and neuronal re-growth [30,37,38]. Cytoskeletal proteins impact variably on tissue survival. Loss of neurofilaments like the microtubule-associated proteins (*Map2*) for example, prevents neurotoxic protein aggregates disrupting axonal transport, whilst vimentin up-regulation reduces the protracted release from macrophages of toxic reactive oxygen species (ROS) [37,38,40,41]. A reduction in Ca2+ ATPase activity in the injured tissue causes a neurotoxic increase in intracellular calcium and up-regulation of genes modulating cell cycle mostly resulting in neuronal death. In contrast, down-regulation of genes involved in neurotransmission via regulation of sodium and potassium channels as well as AMPA receptors exert an anti-apoptotic effect [30-32,39,42-48]. The change of neurons and axons membrane excitability has been associated to neuronal degeneration in animal models and in neurophysiological studies conducted on patients with ALS [49,50]. The predominant down-regulation of these signals may thus be seen to play a part in the cell-survival drive.

Delayed molecular responses, mostly identified distally from the injury epicentre, involve the differential regulation of late *injury genes *which modulate apoptosis, growth, neurotransmission, the homeostasis of the extracellular matrix and of cell metabolism (Figure 2) [28,31,34]. Some of these late responses can have an effect on lipid metabolism. For example, the differential regulation of glycerol-3-phosphate dehydrogenase *(Gpd1)*, a mitochondrial enzyme bridging carbohydrate and lipid metabolism, reduces ROS generation whilst the dioxygenase 12-lipoxygenase *(Alox12) *may work along the same lines, incorporating oxygen into specific positions of polyunsaturated fatty acids [31,51,52]. The down-regulation of anti-apoptotic genes such as *cat*, *Bag1 *and *Bcl2 *can exert an increase in neuronal cell death [53]. As already mentioned, the late activation of key modulators of membrane excitability also reported to become over-expressed in ALS, such as the hyperpolarization-activated cyclic nucleotide-gated cation channel *(Hcn)*, is likely to impair the functional recovery by enhancement of axonal excitability [54] Similarly to growth factors, heat shock proteins exert a neurorestorative effect for neurons, glial and muscle cells, both as a rapid and as a delayed response [32,35,37-39,55].

### Increased vulnerability to spinal trauma: what does it hide?

The degree of tissue destruction and the residual neurological disability following SCI depend primarily on the nature of the mechanical stress (e.g. penetrating injuries versus compressive and/or traction type of impact) [29-39], on the different spatial distributions and temporal activations that different neurorestorative and neurodestructive molecular signals may have, in line with those reported in animal models of SCI (Figures 1 and 2). Certain states modify the response to SCI, including a) the pre-existence of a subclinical neurodegenerative process, a situation that becomes more likely with aging, and b) the presence of a specific genetic trait which increases the vulnerability to trauma.

#### Neurodegeneration and the effects of trauma

Acute and chronic traumatic encephalopathies in collision sports have been linked to the deposition of TAR DNA-binding protein 43 (*Tardbp) *in the brain, a hallmark of ALS pathology, in individuals who will later be affected by a neuromuscular disorder indistinguishable from ALS [13]. Hence, neurotrauma could initiate an ALS-like neuropathology or worsen a pre-existing sub-clinical ALS state. This concept has been investigated using pre-symptomatic rodent models of ALS, engineered using the mutant human superoxide dismutase 1 *(SOD1) *gene which is found in up to 10% of familial cases of ALS [28,56,57]. Both pre-symptomatic *SOD1* mutated rats and mice showed a poor post-injury locomotor recovery, compared to wild type littermates, following mild compression SCI and sciatic nerve injury respectively [28,56]. In the post-injury phase, the transgenic rat cord displayed a more robust activation of several pro-apoptotic genes, cytochrome-C release, a high level of expression of neurofilaments and an early activation of a wide range of inflammatory signals [28]. It has also been possible to identify a significant activation of molecules involved in lipid metabolism, in isoprenoid biosynthesis and in the proteasome ubiquination system, along with a late up-regulation of lysosomal cysteine proteases and of genes involved in neurotransmission. A more subdued surge of growth-promoting signals at the epicenter of injury is another characteristic of the injured transgenic *SOD1 *spinal cord [28,58-61]. Whilst the post-injury transgenic spinal cord displays an altered transcriptional profile compared to wild type tissue, there are no overt histopathological differences between these tissues with regard to the extension of myelin destruction, motor cell loss and the inflammatory infiltrates caudal to the epicenter of injury [28]. This observation illustrates how SCI in pre-symptomatic animals carrying a *SOD1 *gene mutation may not necessarily cause more structural changes compared to wild type animals, although the trauma may be disruptive enough at a molecular level to instigate functional disruption.

#### Aging and SCI

Elderly patients have a 5 to 8-fold higher mortality rate following SCI compared to younger patients [62-66]. The vulnerability to SCI in the elderly may be linked to a process of senescence of the brain, involving beta amyloid deposition in neurons and microglia [67-69]. Aging is also one of the most important risk factors for the development of most neurodegenerative disorders, which manifest clinically after the progressive accumulation of microscopic tissue alterations in the CNS has overcome a certain threshold. Acute or chronic traumatisms may accelerate this process of abnormal protein deposition, leading to the premature surfacing of neurodegenerative conditions. Trauma to the neuroaxis can also enhance the level of protein aggregation, a process that causes the appearance of the histological hallmarks of idiopathic and genetically induced neurodegenerative disorders [40,70,71]. The spectrum of protein aggregates observed in neurodegenerative disorders whose expression could be conditioned by trauma includes beta amyloid and phosphorylated tau proteins normally observed within neurofibrillary tangles in Alzheimer's disease [72], alpha-synuclein within Lewy bodies found in Parkinson's disease [73], neurofilaments in bunina and spheroids bodies typical of ALS neuropathology and prion protein in Prion disease [71]. Trauma may further impair axonal transport and the functioning of the proteasome system, two molecular functions at the origin of the formation of most toxic protein aggregates.

#### Genes modifying the molecular response to trauma

**Figure 3:**
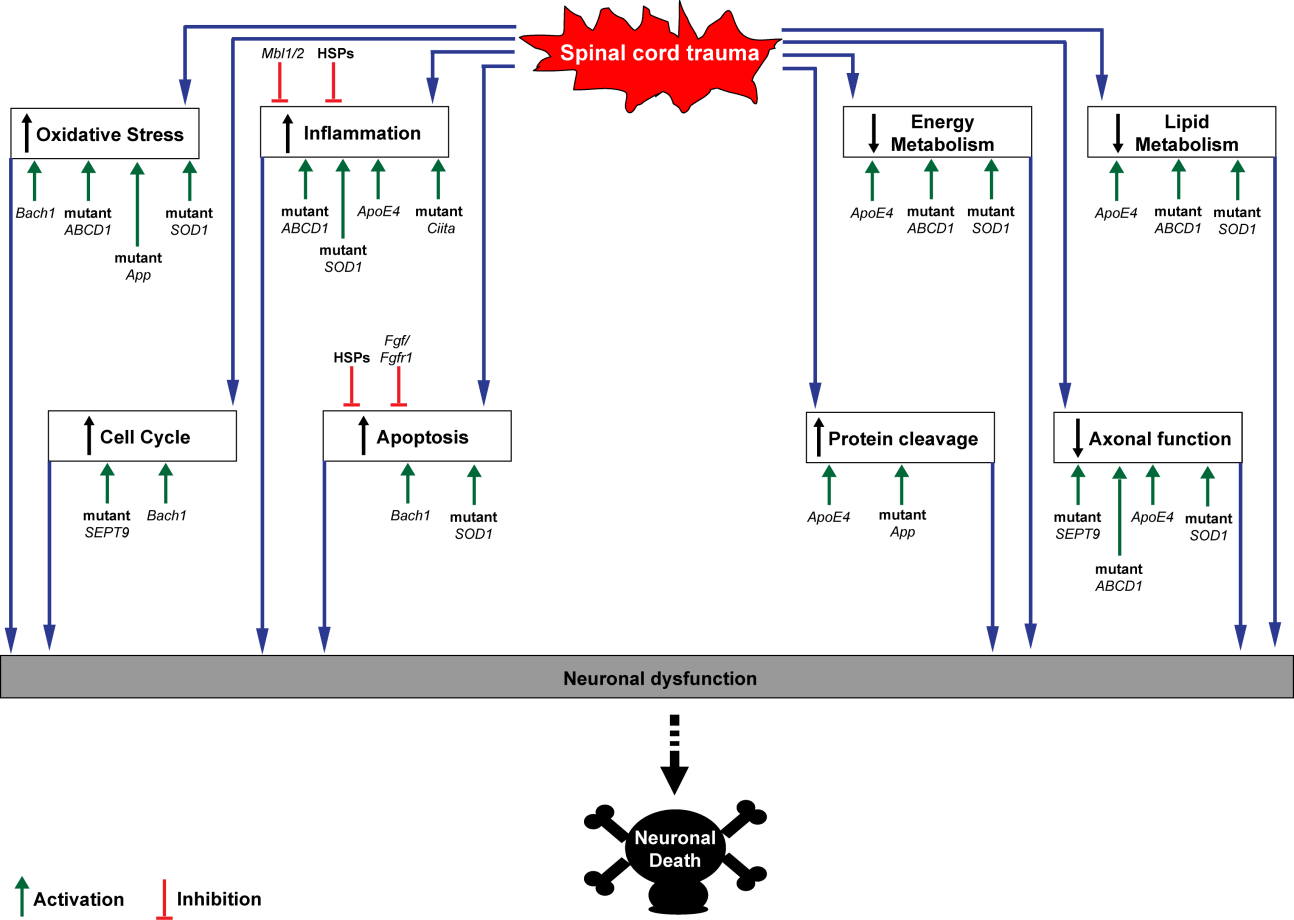
**Schematic diagram showing the cascade of molecular changes initiated by an injury to the spinal cord, which leads to neuronal dysfunction and death**. Those genes thought to be able to modify the effect of trauma (Table 1) have the potential to alter each of the reported molecular pathways, by inhibition (red line) or by activation of a specific response (green arrow), thus changing the overall molecular profile in the injured tissue and affecting the rate of neuronal death.

Recent experimental data show how a number of genes may act as modifiers of animals and humans response to SCI, thus collectively or independently increasing one's susceptibility to injury (Table 1 and Figure 3). Allelic variants of these genes or mutations causing loss or gain of function condition the unraveling of various molecular cascades which are key components of the response to injury (Figure 3). An altered protein cleavage, one of the main driving forces behind protein aggregation in neurodegenerative disorders, can be further enhanced by trauma in the presence of specific Apolipoprotein E (Apoe) and beta amyloid precursor protein (App) variants. The *ApoE4 *allele has been unanimously linked to an increased risk of late onset Alzheimer's disease and to the development of other neurodegenerative disorders with professional boxing [74]. Loss of Apoe reduces recovery following neurotrauma or ischemic insults, as shown in *Apoe*-deficient mice whereas carriers of the *ApoE4 *allele have also a 4 to 6-fold increased risk of developing cervical spondylotic myelopathy (CSM) in a situation of chronic spinal cord compression [2,75]. *Apoe *fragments produced by the trauma-induced proteolytic cleavage of this protein may disrupt the cell's cytoskeleton by phosphorylation of tau and promote neurofibrillary tangles which ultimately cause neuronal death [76,77]. Hence the detrimental effect of the *ApoE4 *allele in neurodegeneration may be partly due to its higher susceptibility to proteolytic cleavage compared to E2 or E3 isoforms [78]. Similarly, a derangement of proteolysis may explain the increased level of deposition of beta amyloid following trauma, as demonstrated neuropathologically in humans and transgenic mice (Tg2576) expressing mutant human beta amyloid precursor protein [52,72,79].

**Table 1:** Gene modifiers of the response to spinal cord injury and/or to neurotrauma.

Gene variant	Experimental paradigm	Gene function	Neuropathology	References
ApoE	Human and ApoE -/- animal models	Lipid transport	Neurofibrillary tangles	Jordan et al., 1997 Saunders et al., 1993 Setzer et al., 2008

ABCD1	Human carriers	Transport and metabolise very long-chain fatty acids	Axonal demylination	Berger & Gartner, 2006 Fatemi et al., 2003 Raymond et al., 2010

Bach1	Bach -/- mice	Pro-oxidant; (transcriptional repressor of heme oxygenase-1)	Cellular death	Kanno et al., 2009 Yamada et al., 2008

SOD1	Animal models	Oxidative stress, apoptosis, inflammation, neurofilaments, lipid metabolism	Neuronal death, Reactive astrogliosis	Jokic et al., 2010 Sharp et al., 2005

TDP-43	Human trauma and Nerve injury animal models	DNA, RNA and protein binding	Neuronal death	Mckee et al., 2010 Moisse et al., 2009

SEPT9	Humans	Cytoskeleton, cell division, tumorigenesis	Axonal degeneration	Kuhlenbaumer et al., 2005

MHC2TA	Root avulsion animal models	Major MHC class II	Neuronal death	Harnesk et al., 2008 Piehl et al., 2007

Beta App	Human trauma and injury animal models	Protein cleavage, oxidative stress	Neurofibrillary tangles	Li et al., 1995 Uryu et al., 2002 Uryu et al., 2007

FGF	Dominant negative animals	Angiogenesis, wound healing, embryonic development	Cellular death	Eckenstein et al., 2006

HSP	Animal models	Molecular chaperones, oxidative stress, apoptosis	Neuronal death	Reddy et al., 2008

The table details for each gene, the experimental context where the effect of the gene was tested and the molecular pathways through to be implicated in the specific gene-driven molecular response to trauma.

Change in lipid metabolism and in the homeostasis of lipid mediators is another route through which genes are thought to modulate the nervous tissue susceptibility to trauma, similarly to what was previously discussed for the *SOD1 *gene. For example, beta amyloid is known to modulate lipid peroxidation whilst Apoe is a lipid-binding protein that is important in the redistribution of lipids among cells in the CNS and in cholesterol transport [72,74,78-80] . Mutations of the ATP-binding cassette transporter subfamily D1 *(ABCD1) *gene, which encode for defective protein transporters in the peroxisomal membrane, affect the homeostasis of saturated and unbranched very long-chain fatty acids. Traumas can precipitate X-linked adrenoleukodystrophy (X-ALD) in young males or a milder variant of this condition named adrenomyeloneuropathy (AMN) in *ABCD1 *mutation carriers, a slowly progressive axonopathy in the spinal cord tracts and in the peripheral nerves [81-84]. In some cases, neuroimaging has shown how the pathological expression of the disease following trauma remains confined to the CNS and to the spinal cord areas more directly affected by the mechanical injury [83,85-89].

Several of the genes mentioned above are likely to modulate tissue vulnerability to mechanical trauma through oxidative stress, an important determinant of SCI-induced secondary injury neuronal loss. X-ALD post mortem brains [90] and mouse model of X-ALD [91] show significant levels of oxidative damage. Mice deficient in Bach1, a transcriptional repressor of the heme oxygenase-1 *(Hmox1) *gene which has a cytoprotective and anti-oxidant effect, showed a better profile of functional recovery following moderate SCI and a significant smaller area of injury [92,93]. *ABCD1 *can also give rise to inflammatory-related demyelination [81]. Trauma-induced lipid peroxidation in mutant *App *animals is also pointing towards oxidative stress as well as a deranged lipid metabolism as important factors in the determination of susceptibility to trauma [79].

Variants of genes exerting control over the inflammatory response, like polymorphisms of the *Ciita (alternative symbol: Mhc2ta) *have been reported to be associated with both lower expression of MHC class II-associated genes and with an increased neurodestruction in animal models of root avulsion injury [94,95]. *ApoE4 *increases the inflammatory tone following neurotrauma with a significant surge of Il6, Tnf and nitric oxide in the injured tissue [96]. Heat shock proteins (HSPs) are intracellular stress-responsive molecular chaperons, which participate in the secondary injury phase by scavenging damaged proteins. Whilst universally known to provide an effective clearance of abnormal proteins, their recognized motor-cell sparing effect in SCI effect is linked to their ability to prevent chronic inflammation, once these proteins are released by acutely stressed microglial, endothelial, and ependymal cells, [97].

The silencing or ablation in dominant-negative animals of the fibroblast growth factor receptor (*Fgfr*), which is known to inhibit fibroblast growth factor (Fgf) signaling, does not appear to cause any overt neurological disorder. However, this genetic manipulation seems to induce a higher level of neuronal vulnerability to a stab injury to the spinal cord in mice [98]. Endogenous Fgf released by astrocytes and neurons after mechanical injury is thought to counteract the excitotoxic or ischemic damage by activating anti-apoptotic signals in stressed neurons [98].

Mannose binding lectin (*Mbl1/2*)-deficient mice have been described to show exacerbated CA3 cell death and remarkable behavioral changes after traumatic brain injury, compared to wild type mice [99]. Mannose binding lectin is a glycoprotein of the collectin family that plays an important role in the host's initial response to infection by initiating complement activation and promoting phagocytosis by leukocytes [100]. The septin-9 *(SEPT9) *gene has been associated to an increased susceptibility to develop a form of brachial plexus pathology as a result of different stressors including immunizations and traumas [3]. *SEPT9 *belongs to the septin family of proteins, GTPases active on cell cycle and on cytoskeletal components, including microtubules and actin [3].

## Conclusions: SCI and the molecular point of no return

The neurological impairment induced by SCI may gradually subside or, despite comprehensive rehabilitative efforts over a period of time, turn into an irreversible functional deficits. More atypical post-injury clinical pictures include localized, non-progressive as well as diffused and evolving forms of amyotrophy, neurological pictures very close to what observed in MND [101]. In some other cases, protracted and repetitive mechanical stress like the strenuous use of a limb due to particular occupational exposures or professional sports have been linked to the development of recurrent painful brachial plexus neuropathies, with features of muscle weakness and atrophy as well as sensory loss, similarly to what seen in hereditary neuralgic amyotrophies [3]. Whether permanent or progressive, the neurological consequences of trauma reflect a complex interplay of genetic and environmental factors, which condition an individual's susceptibility to withstand injury. This paper has embraced the body of experimental data describing genes which may potentially modulate susceptibility to trauma, in order to dissect those molecular events that may be responsible of the establishment of irreversible neurodegeneration in the post-injury phase, here defined as the "point of no return".

We postulate that the response of each individual to injury may operate according to a "molecular threshold", beyond which the response to a particular type of SCI leads to relentless tissue destruction and functional loss. The relatively few studies that have developed an experimental strategy to explore this concept have shown that the genetic determinants likely to be involved in this "fatal switch" modulate inflammation and oxidative stress, participate in lipid metabolism, protein cleavage and in neurofilaments homeostasis, whilst altering the balance between apoptotic and growth signals (Figure 3). It is likely that the contribute of the reported gene modifiers through the molecular pathways activated in injured tissue and their effect in defining the final outcome of SCI, rely on an altered profile of expression of most of the components of these molecular cascades and also to the change of their spatio-temporal regulation with regard to the time and site of injury. For example, a *SOD1 *gene mutation in a pre-symptomatic rat exposed to compression SCI changes significantly the unravelling of molecular events in the first week following the trauma, resulting in a more robust inflammatory response occurring sooner after the impact. The injured tissue neurofilaments heavy chain expression does not decline significantly and the activation of genes involved in lipid metabolism does take place sooner and in a much bigger scale, compared to wild type littermates under the same experimental conditions [28]. The first two events mentioned above are likely to have a detrimental effect through the increased inflammatory and apoptosis-mediated cell destruction and through a surge of cytoskeletal protein aggregation undermining axonal transport, whereas the third event would likely promote cell survival. The post-injury change of homeostasis of lipid and inflammatory mediators, as well as of neurofilaments are examples of complex molecular signals involved in the modulation of irreversible neurodegeneration in different pathological contexts, particularly in ALS [102,103]. The up-regulation of lipids in the post-injury phase is in line with what has been reported in ALS patients and in animal models of ALS, where an early derangement of mediators of lipid homeostasis is a distinctive feature of the pathology and may be part of a rescue mechanism of degenerating neurons [102].

The modality of mechanical force applied to the spinal cord and the level of tissue penetration account also for the different post-injury behaviour in the same *SOD1 *gene mutated rat model. Compression and stabbing spinal cord injuries on the pre-symptomatic G93A-SOD1 rat model of ALS, for example, evoke completely different tissue responses at both molecular and cellular level [28,57]. Surviving motor neurons in the G93A-SOD1 rodents subjected to compression SCI undergo significant atrophy when compared to wild type littermates, a feature not seen using penetrating injuries in the same animal model [28,57].

Both in animal models of most neurodegenerative disorders and in real life, neurotrauma may precipitate the pathological process which is already altering the fine structure and the function of a macroscopically intact tissue. The injury may simply accelerate the course of neurodegeneration, which would have otherwise followed a different time line. Aging is clearly an important factor in this interaction, as it is an important risk factor for the development of neurodegenerative disorders and of the subtle molecular changes that pre-date the main clinical manifestations of most neurological conditions.

Understanding the molecular framework of the response to SCI in relationship to aging and to the presence of a potential underlying genetic vulnerability is an essential precondition for the development of disease-modifying treatments, of prognostic biomarkers and to monitor the response to a targeted and timely treatment strategy. A better knowledge of the molecular framework which conditions the outcome from neurotrauma is also an ideal ground for a better understanding of the wider concept of both idiopathic and genetically-induced neurodegeneration.

## List of abbreviations

ALS: Amyotrophic lateral sclerosis; ALD: Adrenoleukodystrophy; *Alox12*: 12-Lipoxygenase; AMN: Adrenomyeloneuropathy; Apoe: Apolipoprotein; *ApoE4*: Apolipoprotein E4 allele; *App*: Amyloid beta (A4) precursor protein; *Atp1a3*: ATPase, Na+/K+ transporting, alpha 3 polypeptide; *Atp2a1*: ATPase, Ca++ transporting, cardiac muscle, fast twitch 1; *Atp2b2*: ATPase, Ca++ transporting, plasma membrane 2; *Bad*: Bcl2 associated agonist of cell death; *Bach1*: BTB and CNC homology 1, basic leucine zipper transcription factor 1; *Bag1*: Bcl2-associated athanogene; *Bcl2*: B-cell CLL/lymphoma 2; *Bdnf*: Brain-derived neurotrophic factor; *Bmp2*: Bone morphogenetic protein 2; *Bmpr1a*: Bone morphogenetic protein receptor, type IA; *Cat*: Catalase; *Ccnd1*: cyclin D1; *Ciita*: Class II, major histocompatibility complex, transactivator; CSM: Cervical spondylotic myelopathy; *Dcn*: Decorin; fgf: Fibroblast growth factor; *Fgfr1*: Fibroblast growth factor receptor 1; GABA: gamma-aminobutyric acid; *Gabbr1*: GABA B receptor; *Gabra5*: GABA A receptor, alpha 5; *Gabrb1*: GABA A receptor, beta 1 *Gabbr2*: GABA B receptor 2; *Gadd45a *Growth arrest and DNA-damage-inducible gene 45a; *Gpd1*: Glycerol-3-phosphate dehydrogenase 1; *Gria3*: ionotropic glutamate receptor 3; *Grm3*: Metabotropic glutamate receptor 3; *Hcn2*: Hyperpolarization activated cyclic nucleotide-gated potassium channel 2; *Hmox1*: Heme oxygenase (decycling) 1; HSPs: Heat shock proteins; *Hspb1*: Heat shock 27kDa protein 1; *Hspa4*: Heat shock protein 70KDa protein 4; *Igf1*: Insulin-like growth factor 1; *Il1b*: Interleukin 1 beta; *Il6*: Interleukin 6; *Kcnc1*: Potassium voltage gated channel, Shaw-related subfamily, member 1; *Kcnh2*: Potassium voltage-gated channel, subfamily H (eag-related), member 2; *Kcnk1*: Potassium channel, subfamily K, member 1; *Map2*: Microtubule-associated protein 2; *Mbl1/2*: Mannose-binding lectin (protein A and C) 1 and 2; MND: Motor neurons disease; MMP: Matrix metalloproteinase; *Mt1a*: Metallothionein 1a*; Mt2*: Metallothionein II; *Myc*: Myelocytomatosis oncogene; *Nefl*: Neurofilament light polypeptide; *Ngfr*: Low-affinity nerve growth factor; *Ntrk2*; Neurotrophic tyrosine kinase receptor type 2; *Pcna*: Proliferating cell nuclear antigen; ROS: Reactive oxygen species; *SEPT9*; Septin 9; *Scn1a*: sodium channel, voltage-gated, type I, alpha; *Scn8a*: odium channel, voltage gated, type VIII, alpha subunit; *Slc6a1*: solute carrier family 6 (neurotransmitter transporter, GABA), member 1; *SOD1*: Superoxide dismutase 1; *Tardbp*: TAR DNA binding protein; *Tnf*: Tumor necrosis factor; *Vim*: Vimentin.

## Competing interests

The authors declare that they have no competing interests.

## Authors' contributions

PKY & AM: Equal contribution to the writing of this manuscript and approval of the final submitted text.

## References

[B1] Ghatak NR Campbell WW Lippman RH Hadfield MG Anterior horn changes of motor neuron disease associated with demyelinating radiculopathy J Neuropathol Exp Neurol 1986 45 385 395 10.1097/00005072-198607000-00001 3014067

[B2] Setzer M Hermann E Seifert V Marquardt G Apolipoprotein E gene polymorphism and the risk of cervical myelopathy in patients with chronic spinal cord compression Spine (Phila Pa 1976) 2008 33 497 502 10.1097/BRS.0b013e3181657cf7 18317192

[B3] Kuhlenbaumer G Hannibal MC Nelis E Schirmacher A Verpoorten N Meuleman J Watts GD De VE Young P Stogbauer F Mutations in SEPT9 cause hereditary neuralgic amyotrophy Nat Genet 2005 37 1044 1046 10.1038/ng1649 16186812

[B4] Leigh PN Abrahams S Al-Chalabi A Ampong MA Goldstein LH Johnson J Lyall R Moxham J Mustfa N Rio A The management of motor neurone disease J Neurol Neurosurg Psychiatry 2003 74 Suppl 4 iv32 iv47 14645465 10.1136/jnnp.74.suppl_4.iv32 PMC1765641

[B5] Abel EL Football increases the risk for Lou Gehrig's disease, amyotrophic lateral sclerosis Percept Mot Skills 2007 104 1251 1254 17879657 10.2466/pms.104.4.1251-1254

[B6] Binazzi A Belli S Uccelli R Desiato MT Talamanca IF Antonini G Corsi FM Scoppetta C Inghilleri M Pontieri FE An exploratory case-control study on spinal and bulbar forms of amyotrophic lateral sclerosis in the province of Rome Amyotroph Lateral Scler 2009 10 361 369 10.3109/17482960802382313 19922125

[B7] Bracco L Antuono P Amaducci L Study of epidemiological and etiological factors of amyotrophic lateral sclerosis in the province of Florence, Italy Acta Neurol Scand 1979 60 112 124 495044 10.1111/j.1600-0404.1979.tb02958.x

[B8] Chen H Richard M Sandler DP Umbach DM Kamel F Head injury and amyotrophic lateral sclerosis Am J Epidemiol 2007 166 810 816 10.1093/aje/kwm153 17641152 PMC2239342

[B9] Chio A Benzi G Dossena M Mutani R Mora G Severely increased risk of amyotrophic lateral sclerosis among Italian professional football players Brain 2005 128 472 476 10.1093/brain/awh373 15634730

[B10] Chio A Calvo A Dossena M Ghiglione P Mutani R Mora G ALS in Italian professional soccer players: the risk is still present and could be soccer-specific Amyotroph Lateral Scler 2009 10 205 209 10.1080/17482960902721634 19267274

[B11] Kondo K Tsubaki T Case-control studies of motor neuron disease: association with mechanical injuries Arch Neurol 1981 38 220 226 10.1001/archneur.1981.00510040046007 7011280

[B12] Matser JT Kessels AG Lezak MD Troost J A dose-response relation of headers and concussions with cognitive impairment in professional soccer players J Clin Exp Neuropsychol 2001 23 770 774 10.1076/jcen.23.6.770.1029 11910543

[B13] McKee AC Gavett BE Stern RA Nowinski CJ Cantu RC Kowall NW Perl DP Hedley-Whyte ET Price B Sullivan C TDP-43 proteinopathy and motor neuron disease in chronic traumatic encephalopathy J Neuropathol Exp Neurol 2010 69 918 929 10.1097/NEN.0b013e3181ee7d85 20720505 PMC2951281

[B14] Riggs JE Antecedent trauma and amyotrophic lateral sclerosis in young adult men Mil Med 1993 158 55 57 8437741

[B15] Riggs JE The latency between traumatic axonal injury and the onset of amyotrophic lateral sclerosis in young adult men Mil Med 2001 166 731 732 11515328

[B16] Schmidt S Kwee LC Allen KD Oddone EZ Association of ALS with head injury, cigarette smoking and APOE genotypes J Neurol Sci 2010 291 22 29 10.1016/j.jns.2010.01.011 20129626 PMC2840700

[B17] Strickland D Smith SA Dolliff G Goldman L Roelofs RI Physical activity, trauma, and ALS: a case-control study Acta Neurol Scand 1996 94 45 50 8874593 10.1111/j.1600-0404.1996.tb00038.x

[B18] Wicks P Ganesalingham J Collin C Prevett M Leigh NP Al-Chalabi A Three soccer playing friends with simultaneous amyotrophic lateral sclerosis Amyotroph Lateral Scler 2007 8 177 179 10.1080/17482960701195220 17538780

[B19] Kihira T Kanno S Miwa H Okamoto K Kondo T The role of exogenous risk factors in amyotrophic lateral sclerosis in Wakayama, Japan Amyotroph Lateral Scler 2007 8 150 156 10.1080/17482960601179407 17538776

[B20] Yamada M Furukawa Y Hirohata M Amyotrophic lateral sclerosis: frequent complications by cervical spondylosis J Orthop Sci 2003 8 878 881 10.1007/s00776-003-0712-0 14648282

[B21] Bareyre FM Schwab ME Inflammation, degeneration and regeneration in the injured spinal cord: insights from DNA microarrays Trends Neurosci 2003 26 555 563 10.1016/j.tins.2003.08.004 14522149

[B22] DeWitt DS Prough DS Taylor CL Whitley JM Reduced cerebral blood flow, oxygen delivery, and electroencephalographic activity after traumatic brain injury and mild hemorrhage in cats J Neurosurg 1992 76 812 821 10.3171/jns.1992.76.5.0812 1564544

[B23] Kruman II Mattson MP Pivotal role of mitochondrial calcium uptake in neural cell apoptosis and necrosis J Neurochem 1999 72 529 540 9930724 10.1046/j.1471-4159.1999.0720529.x

[B24] Pedersen MO Jensen R Pedersen DS Skjolding AD Hempel C Maretty L Penkowa M Metallothionein-I+II in neuroprotection Biofactors 2009 35 315 325 10.1002/biof.44 19655389

[B25] Takahashi H Manaka S Sano K Changes in extracellular potassium concentration in cortex and brain stem during the acute phase of experimental closed head injury J Neurosurg 1981 55 708 717 10.3171/jns.1981.55.5.0708 7310492

[B26] Yamakami I McIntosh TK Effects of traumatic brain injury on regional cerebral blood flow in rats as measured with radiolabeled microspheres J Cereb Blood Flow Metab 1989 9 117 124 10.1038/jcbfm.1989.16 2910893

[B27] Zemper ED Analysis of cerebral concussion frequency with the most commonly used models of football helmets J Athl Train 1994 29 44 50 16558258 PMC1317758

[B28] Jokic N Yip PK Michael-Titus A Priestley JV Malaspina A The human G93A-SOD1 mutation in a pre-symptomatic rat model of amyotrophic lateral sclerosis increases the vulnerability to a mild spinal cord compression BMC Genomics 2010 11 633 10.1186/1471-2164-11-633 21078175 PMC3020590

[B29] Malaspina A Jokic N Huang WL Priestley JV Comparative analysis of the time-dependent functional and molecular changes in spinal cord degeneration induced by the G93A SOD1 gene mutation and by mechanical compression BMC Genomics 2008 9 500 10.1186/1471-2164-9-500 18947433 PMC2585103

[B30] Aimone JB Leasure JL Perreau VM Thallmair M Spatial and temporal gene expression profiling of the contused rat spinal cord Exp Neurol 2004 189 204 221 10.1016/j.expneurol.2004.05.042 15380473

[B31] Bareyre FM Haudenschild B Schwab ME Long-lasting sprouting and gene expression changes induced by the monoclonal antibody IN-1 in the adult spinal cord J Neurosci 2002 22 7097 7110 12177206 10.1523/JNEUROSCI.22-16-07097.2002 PMC6757902

[B32] Carmel JB Galante A Soteropoulos P Tolias P Recce M Young W Hart RP Gene expression profiling of acute spinal cord injury reveals spreading inflammatory signals and neuron loss Physiol Genomics 2001 7 201 213 11773606 10.1152/physiolgenomics.00074.2001

[B33] Di Giovanni S Knoblach SM Brandoli C Aden SA Hoffman EP Faden AI Gene profiling in spinal cord injury shows role of cell cycle in neuronal death Ann Neurol 2003 53 454 468 10.1002/ana.10472 12666113

[B34] Fan M Mi R Yew DT Chan WY Analysis of gene expression following sciatic nerve crush and spinal cord hemisection in the mouse by microarray expression profiling Cell Mol Neurobiol 2001 21 497 508 10.1023/A:1013867306555 11860187 PMC11533824

[B35] Nesic O Svrakic NM Xu GY McAdoo D Westlund KN Hulsebosch CE Ye Z Galante A Soteropoulos P Tolias P DNA microarray analysis of the contused spinal cord: effect of NMDA receptor inhibition J Neurosci Res 2002 68 406 423 10.1002/jnr.10171 11992467

[B36] Pan JZ Ni L Sodhi A Aguanno A Young W Hart RP Cytokine activity contributes to induction of inflammatory cytokine mRNAs in spinal cord following contusion J Neurosci Res 2002 68 315 322 10.1002/jnr.10215 12111861

[B37] Resnick DK Schmitt C Miranpuri GS Dhodda VK Isaacson J Vemuganti R Molecular evidence of repair and plasticity following spinal cord injury Neuroreport 2004 15 837 839 10.1097/00001756-200404090-00020 15073526

[B38] Schmitt C Miranpuri GS Dhodda VK Isaacson J Vemuganti R Resnick DK Changes in spinal cord injury-induced gene expression in rat are strain-dependent Spine J 2006 6 113 119 10.1016/j.spinee.2005.05.379 16517380

[B39] Song G Cechvala C Resnick DK Dempsey RJ Rao VL GeneChip analysis after acute spinal cord injury in rat J Neurochem 2001 79 804 815 11723173 10.1046/j.1471-4159.2001.00626.x

[B40] Lin H Schlaepfer WW Role of neurofilament aggregation in motor neuron disease Ann Neurol 2006 60 399 406 10.1002/ana.20965 17006927

[B41] Mor-Vaknin N Punturieri A Sitwala K Markovitz DM Vimentin is secreted by activated macrophages Nat Cell Biol 2003 5 59 63 10.1038/ncb898 12483219

[B42] Lees GJ Inhibition of sodium-potassium-ATPase: a potentially ubiquitous mechanism contributing to central nervous system neuropathology Brain Res Brain Res Rev 1991 16 283 300 1665097 10.1016/0165-0173(91)90011-v

[B43] Noh KM Yokota H Mashiko T Castillo PE Zukin RS Bennett MV Blockade of calcium-permeable AMPA receptors protects hippocampal neurons against global ischemia-induced death Proc Natl Acad Sci USA 2005 102 12230 12235 10.1073/pnas.0505408102 16093311 PMC1189338

[B44] Palmer AM Carter N The role of sodium channels in disease Drug News Perspect 2001 14 568 576 10.1358/dnp.2001.14.9.858413 12806444

[B45] Spillson AB Russell JW Metabotropic glutamate receptor regulation of neuronal cell death Exp Neurol 2003 184 Suppl 1 S97 105 14597332 10.1016/j.expneurol.2003.08.001

[B46] Yu SP Yeh CH Sensi SL Gwag BJ Canzoniero LM Farhangrazi ZS Ying HS Tian M Dugan LL Choi DW Mediation of neuronal apoptosis by enhancement of outward potassium current Science 1997 278 114 117 10.1126/science.278.5335.114 9311914

[B47] Zeevalk GD Nicklas WJ Attenuation of excitotoxic cell swelling and GABA release by the GABA transport inhibitor SKF 89976A Mol Chem Neuropathol 1996 29 27 36 10.1007/BF02815191 8887938

[B48] Zeevalk GD Nicklas WJ Activity at the GABA transporter contributes to acute cellular swelling produced by metabolic impairment in retina Vision Res 1997 37 3463 3470 10.1016/S0042-6989(97)00184-3 9425523

[B49] Meehan CF Moldovan M Marklund SL Graffmo KS Nielsen JB Hultborn H Intrinsic properties of lumbar motor neurones in the adult G127insTGGG superoxide dismutase-1 mutant mouse in vivo: evidence for increased persistent inward currents Acta Physiol (Oxf) 2010 200 361 376 10.1111/j.1748-1716.2010.02188.x 20874803

[B50] Shibuya K Misawa S Arai K Nakata M Kanai K Yoshiyama Y Ito K Isose S Noto Y Nasu S Markedly reduced axonal potassium channel expression in human sporadic amyotrophic lateral sclerosis: An immunohistochemical study Exp Neurol 2011 232 149 153 10.1016/j.expneurol.2011.08.015 21906595

[B51] Brand MD The sites and topology of mitochondrial superoxide production Exp Gerontol 2010 45 466 472 10.1016/j.exger.2010.01.003 20064600 PMC2879443

[B52] Li Y Maher P Schubert D A role for 12-lipoxygenase in nerve cell death caused by glutathione depletion Neuron 1997 19 453 463 10.1016/S0896-6273(00)80953-8 9292733

[B53] Pedersen MO Larsen A Stoltenberg M Penkowa M Cell death in the injured brain: roles of metallothioneins Prog Histochem Cytochem 2009 44 1 27 10.1016/j.proghi.2008.10.002 19348909

[B54] Chu HY Zhen X Hyperpolarization-activated, cyclic nucleotide-gated (HCN) channels in the regulation of midbrain dopamine systems Acta Pharmacol Sin 2010 31 1036 1043 10.1038/aps.2010.105 20676119 PMC4002296

[B55] Brown IR Heat shock proteins and protection of the nervous system Ann N Y Acad Sci 2007 1113 147 158 10.1196/annals.1391.032 17656567

[B56] Sharp PS Dick JR Greensmith L The effect of peripheral nerve injury on disease progression in the SOD1(G93A) mouse model of amyotrophic lateral sclerosis Neuroscience 2005 130 897 910 10.1016/j.neuroscience.2004.09.069 15652988

[B57] Suzuki M Klein S Wetzel EA Meyer M McHugh J Tork C Hayes A Svendsen CN Acute glial activation by stab injuries does not lead to overt damage or motor neuron degeneration in the G93A mutant SOD1 rat model of amyotrophic lateral sclerosis Exp Neurol 2010 221 346 352 10.1016/j.expneurol.2009.12.004 20005223 PMC2839070

[B58] Ferraiuolo L Heath PR Holden H Kasher P Kirby J Shaw PJ Microarray analysis of the cellular pathways involved in the adaptation to and progression of motor neuron injury in the SOD1 G93A mouse model of familial ALS J Neurosci 2007 27 9201 9219 10.1523/JNEUROSCI.1470-07.2007 17715356 PMC6672214

[B59] Kabashi E Durham HD Failure of protein quality control in amyotrophic lateral sclerosis Biochim Biophys Acta 2006 1762 1038 1050 16876390 10.1016/j.bbadis.2006.06.006

[B60] Kudo LC Parfenova L Vi N Lau K Pomakian J Valdmanis P Rouleau GA Vinters HV Wiedau-Pazos M Karsten SL Integrative gene-tissue microarray-based approach for identification of human disease biomarkers: application to amyotrophic lateral sclerosis Hum Mol Genet 2010 19 3233 3253 10.1093/hmg/ddq232 20530642

[B61] Lobsiger CS Boillee S Cleveland DW Toxicity from different SOD1 mutants dysregulates the complement system and the neuronal regenerative response in ALS motor neurons Proc Natl Acad Sci USA 2007 104 7319 7326 10.1073/pnas.0702230104 17463094 PMC1863491

[B62] Fassett DR Harrop JS Maltenfort M Jeyamohan SB Ratliff JD Anderson DG Hilibrand AS Albert TJ Vaccaro AR Sharan AD Mortality rates in geriatric patients with spinal cord injuries J Neurosurg Spine 2007 7 277 281 10.3171/SPI-07/09/277 17877260

[B63] Furlan JC Fehlings MG The impact of age on mortality, impairment, and disability among adults with acute traumatic spinal cord injury J Neurotrauma 2009 26 1707 1717 10.1089/neu.2009.0888 19413491 PMC2822797

[B64] Jackson AP Haak MH Khan N Meyer PR Cervical spine injuries in the elderly: acute postoperative mortality Spine (Phila Pa 1976) 2005 30 1524 1527 10.1097/01.brs.0000167822.75063.8c 15990667

[B65] Kuhne CA Ruchholtz S Kaiser GM Nast-Kolb D Mortality in severely injured elderly trauma patients--when does age become a risk factor? World J Surg 2005 29 1476 1482 10.1007/s00268-005-7796-y 16228923

[B66] Scivoletto G Morganti B Ditunno P Ditunno JF Molinari M Effects on age on spinal cord lesion patients' rehabilitation Spinal Cord 2003 41 457 464 10.1038/sj.sc.3101489 12883544

[B67] Rodrigue KM Kennedy KM Park DC Beta-amyloid deposition and the aging brain Neuropsychol Rev 2009 19 436 450 10.1007/s11065-009-9118-x 19908146 PMC2844114

[B68] Scheibel ME Lindsay RD Tomiyasu U Scheibel AB Progressive dendritic changes in aging human cortex Exp Neurol 1975 47 392 403 10.1016/0014-4886(75)90072-2 48474

[B69] Streit WJ Sammons NW Kuhns AJ Sparks DL Dystrophic microglia in the aging human brain Glia 2004 45 208 212 10.1002/glia.10319 14730714

[B70] Anderton BH Changes in the ageing brain in health and disease Philos Trans R Soc Lond B Biol Sci 1997 352 1781 1792 10.1098/rstb.1997.0162 9460061 PMC1692130

[B71] Lindner AB Demarez A Protein aggregation as a paradigm of aging Biochim Biophys Acta 2009 1790 980 996 10.1016/j.bbagen.2009.06.005 19527771

[B72] Uryu K Chen XH Martinez D Browne KD Johnson VE Graham DI Lee VM Trojanowski JQ Smith DH Multiple proteins implicated in neurodegenerative diseases accumulate in axons after brain trauma in humans Exp Neurol 2007 208 185 192 10.1016/j.expneurol.2007.06.018 17826768 PMC3979356

[B73] Eller M Williams DR alpha-Synuclein in Parkinson disease and other neurodegenerative disorders Clin Chem Lab Med 2011 49 403 408 10.1515/CCLM.2011.077 21342025

[B74] Jordan BD Relkin NR Ravdin LD Jacobs AR Bennett A Gandy S Apolipoprotein E epsilon4 associated with chronic traumatic brain injury in boxing JAMA 1997 278 136 140 10.1001/jama.1997.03550020068040 9214529

[B75] Jha A Lammertse DP Coll JR Charlifue S Coughlin CT Whiteneck GG Worley G Apolipoprotein E epsilon4 allele and outcomes of traumatic spinal cord injury J Spinal Cord Med 2008 31 171 176 18581664 10.1080/10790268.2008.11760708 PMC2565476

[B76] Strittmatter WJ Weisgraber KH Huang DY Dong LM Salvesen GS Pericak-Vance M Schmechel D Saunders AM Goldgaber D Roses AD Binding of human apolipoprotein E to synthetic amyloid beta peptide: isoform-specific effects and implications for late-onset Alzheimer disease Proc Natl Acad Sci USA 1993 90 8098 8102 10.1073/pnas.90.17.8098 8367470 PMC47295

[B77] Xu Q Walker D Bernardo A Brodbeck J Balestra ME Huang Y Intron-3 retention/splicing controls neuronal expression of apolipoprotein E in the CNS J Neurosci 2008 28 1452 1459 10.1523/JNEUROSCI.3253-07.2008 18256266 PMC6671590

[B78] Mahley RW Apolipoprotein E: cholesterol transport protein with expanding role in cell biology Science 1988 240 622 630 10.1126/science.3283935 3283935

[B79] Uryu K Laurer H McIntosh T Pratico D Martinez D Leight S Lee VM Trojanowski JQ Repetitive mild brain trauma accelerates Abeta deposition, lipid peroxidation, and cognitive impairment in a transgenic mouse model of Alzheimer amyloidosis J Neurosci 2002 22 446 454 11784789 10.1523/JNEUROSCI.22-02-00446.2002 PMC6758680

[B80] Saunders AM Strittmatter WJ Schmechel D George-Hyslop PH Pericak-Vance MA Joo SH Rosi BL Gusella JF Crapper-MacLachlan DR Alberts MJ Association of apolipoprotein E allele epsilon 4 with late-onset familial and sporadic Alzheimer's disease Neurology 1993 43 1467 1472 8350998 10.1212/wnl.43.8.1467

[B81] Berger J Gartner J X-linked adrenoleukodystrophy: clinical, biochemical and pathogenetic aspects Biochim Biophys Acta 2006 1763 1721 1732 10.1016/j.bbamcr.2006.07.010 16949688

[B82] Moser HW Raymond GV Dubey P Adrenoleukodystrophy: new approaches to a neurodegenerative disease JAMA 2005 294 3131 3134 10.1001/jama.294.24.3131 16380594

[B83] Raymond GV Seidman R Monteith TS Kolodny E Sathe S Mahmood A Powers JM Head trauma can initiate the onset of adreno-leukodystrophy J Neurol Sci 2010 290 70 74 10.1016/j.jns.2009.11.005 19945717

[B84] Schaumburg HH Powers JM Raine CS Suzuki K Richardson EP Jr Adrenoleukodystrophy. A clinical and pathological study of 17 cases Arch Neurol 1975 32 577 591 10.1001/archneur.1975.00490510033001 169765

[B85] Carmant L Decarie JC Fon E Shevell MI Transient visual symptoms as the initial manifestation of childhood adrenoleukodystrophy Pediatr Neurol 1998 19 62 64 10.1016/S0887-8994(98)00015-0 9682889

[B86] Fatemi A Barker PB Ulug AM Nagae-Poetscher LM Beauchamp NJ Moser AB Raymond GV Moser HW Naidu S MRI and proton MRSI in women heterozygous for X-linked adrenoleukodystrophy Neurology 2003 60 1301 1307 12707433 10.1212/01.wnl.0000059546.15529.cb

[B87] Turpin JC Paturneau-Jouas M Sereni C Pluot M Baumann N Adult disclosure of a case of familial adrenoleukodystrophy Rev Neurol (Paris) 1985 141 289 295 2990005

[B88] Weller M Liedtke W Petersen D Opitz H Poremba M Very-late-onset adrenoleukodystrophy: possible precipitation of demyelination by cerebral contusion Neurology 1992 42 367 370 1736167 10.1212/wnl.42.2.367

[B89] Wilkinson IA Hopkins IJ Pollard AC Can head injury influence the site of demyelination in adrenoleukodystrophy? Dev Med Child Neurol 1987 29 797 800 3691981 10.1111/j.1469-8749.1987.tb08827.x

[B90] Gilg AG Singh AK Singh I Inducible nitric oxide synthase in the central nervous system of patients with X-adrenoleukodystrophy J Neuropathol Exp Neurol 2000 59 1063 1069 11138926 10.1093/jnen/59.12.1063

[B91] Powers JM Pei Z Heinzer AK Deering R Moser AB Moser HW Watkins PA Smith KD Adreno-leukodystrophy: oxidative stress of mice and men J Neuropathol Exp Neurol 2005 64 1067 1079 10.1097/01.jnen.0000190064.28559.a4 16319717

[B92] Kanno H Ozawa H Dohi Y Sekiguchi A Igarashi K Itoi E Genetic ablation of transcription repressor Bach1 reduces neural tissue damage and improves locomotor function after spinal cord injury in mice J Neurotrauma 2009 26 31 39 10.1089/neu.2008.0667 19119918

[B93] Yamada K Tanaka N Nakanishi K Kamei N Ishikawa M Mizuno T Igarashi K Ochi M Modulation of the secondary injury process after spinal cord injury in Bach1-deficient mice by heme oxygenase-1 J Neurosurg Spine 2008 9 611 620 10.3171/SPI.2008.10.08488 19035757

[B94] Harnesk K Swanberg M Ockinger J Diez M Lidman O Wallstrom E Lobell A Olsson T Piehl F Vra4 congenic rats with allelic differences in the class II transactivator gene display altered susceptibility to experimental autoimmune encephalomyelitis J Immunol 2008 180 3289 3296 18292553 10.4049/jimmunol.180.5.3289

[B95] Piehl F Swanberg M Lidman O The axon reaction: identifying the genes that make a difference Physiol Behav 2007 92 67 74 10.1016/j.physbeh.2007.05.030 17561176

[B96] Colton CA Brown CM Vitek MP Sex steroids, APOE genotype and the innate immune system Neurobiol Aging 2005 26 363 372 10.1016/j.neurobiolaging.2004.08.001 15639315

[B97] Reddy SJ La MF Park P The role of heat shock proteins in spinal cord injury Neurosurg Focus 2008 25 E4 18980478 10.3171/FOC.2008.25.11.E4

[B98] Eckenstein FP McGovern T Kern D Deignan J Neuronal vulnerability in transgenic mice expressing an inducible dominant-negative FGF receptor Exp Neurol 2006 198 338 349 10.1016/j.expneurol.2005.12.020 16487970

[B99] Yager PH You Z Qin T Kim HH Takahashi K Ezekowitz AB Stahl GL Carroll MC Whalen MJ Mannose binding lectin gene deficiency increases susceptibility to traumatic brain injury in mice J Cereb Blood Flow Metab 2008 28 1030 1039 10.1038/sj.jcbfm.9600605 18183030

[B100] Takahashi K Ip WE Michelow IC Ezekowitz RA The mannose-binding lectin: a prototypic pattern recognition molecule Curr Opin Immunol 2006 18 16 23 10.1016/j.coi.2005.11.014 16368230 PMC7126801

[B101] Cerami C Valentino F Piccoli F La BV A cervical myelopathy with a Hirayama disease-like phenotype Neurol Sci 2008 29 451 454 10.1007/s10072-008-1058-3 19057849

[B102] Dupuis L Corcia P Fergani A Gonzalez De Aguilar JL Bonnefont-Rousselot D Bittar R Seilhean D Hauw JJ Lacomblez L Loeffler JP Dyslipidemia is a protective factor in amyotrophic lateral sclerosis Neurology 2008 70 1004 1009 10.1212/01.wnl.0000285080.70324.27 18199832

[B103] Kim SM Kim H Kim JE Park KS Sung JJ Kim SH Lee KW Amyotrophic lateral sclerosis is associated with hypolipidemia at the presymptomatic stage in mice PLoS One 2011 6 e17985 10.1371/journal.pone.0017985 21464953 PMC3064597

